# Supplementary material for: Occurrence and antimicrobial susceptibility of Staphylococcus aureus in dairy farms and personnel in selected towns of West Shewa Zone, Oromia, Ethiopia
Source: PLoS One. 2022 Nov 21;17(11):e0277805. doi: 10.1371/journal.pone.0277805 (PMC9678306; doi:10.1371/journal.pone.0277805)
Supplement: S1 File — (DOCX) [file pone.0277805.s001.docx]

**Supplementary file 1.** **Farmers’ knowledge of factors causing AMR**

| Knowledge question | Agree  N (%) | Uncertain  N (%) | Disagree  N (%) | Total  N (%) | Mean ± SD |
| --- | --- | --- | --- | --- | --- |
| Using over or under dose of antimicrobial in animals or humans causes AMR | 30  40.54 | 28  37.84 | 16  21.62 | 74  100 | 1.19 ± 0.77 |
| Frequently using the same antibiotics in animals and humans have an effect on AMR | 22  29.74 | 36  48.65 | 16  21.62 | 74  100 | 1.08 ± 0.72 |
| Inappropriate use of disinfectants in dairy farms results in the occurrence of AMR | 8  10.81 | 38  51.35 | 28  37.84 | 74  100 | 0.73 ± 0.65 |
| Treating the animals/human own decision causes the AMR | 16  21.6 | 39  52.7 | 19  25.7 | 74  100 | 0.96 ± 0.77 |

N= number, SD = standard deviation
