# Supplementary material for: Occurrence and antimicrobial susceptibility of Staphylococcus aureus in dairy farms and personnel in selected towns of West Shewa Zone, Oromia, Ethiopia
Source: PLoS One. 2022 Nov 21;17(11):e0277805. doi: 10.1371/journal.pone.0277805 (PMC9678306; doi:10.1371/journal.pone.0277805)
Supplement: S2 File — (DOCX) [file pone.0277805.s002.docx]

**Supplementary file 2: The response of the farmers to attitude questions on the factors causing AMR**

| Attitude questions | Agree  N (%) | Uncertain  N (%) | Disagree  N (%) | Total  N (%) | Mean ± SD |
| --- | --- | --- | --- | --- | --- |
| Inappropriate use of antimicrobials causes antimicrobial resistance | 33  44.59 | 25  33.78 | 16  21.62 | 74  100 | 1.23 ± 0.79 |
| Missing one or two doses does not alter the effectiveness of antimicrobials | 25  33.78 | 37  50 | 12  16.22 | 74  100 | 1.16 ± 0.69 |
| Antimicrobial treatment should be stopped as soon as the patient feels better | 24  32.43 | 37  50 | 13  17.57 | 74  100 | 1.15 ± 0.70 |
| I prefer to be able to buy antimicrobials from the pharmacy without a prescription for my animals and family members while they get sick | 32  43.24 | 23  31.08 | 19  25.68 | 74  100 | 1.16 ± 0.82 |
| It is good to be able to get antimicrobials from relatives /friends without being seen by a medical doctor | 22  29.73 | 38  51.35 | 14  18.92 | 74  100 | 1.11 ± 0.69 |
| I always complete the course of treatment with antimicrobials even if I feel better | 27  36.49 | 34  45.95 | 13  17.57 | 74  100 | 1.19 ± 0.72 |
| Keeping leftover antimicrobial drugs for future use is good | 13  17.57 | 28  37.84 | 33  44.59 | 74  100 | 0.72 ± 0.75 |

N= number, SD = standard deviation
