# Supplementary material for: Occurrence and antimicrobial susceptibility of Staphylococcus aureus in dairy farms and personnel in selected towns of West Shewa Zone, Oromia, Ethiopia
Source: PLoS One. 2022 Nov 21;17(11):e0277805. doi: 10.1371/journal.pone.0277805 (PMC9678306; doi:10.1371/journal.pone.0277805)
Supplement: S3 File — (DOCX) [file pone.0277805.s003.docx]

**Supplementary file 3: The response of the farmers to the practice questions on the factors causing AMR**

| Practice questions | Always  N (%) | Sometimes  N (%) | Never  N (%) | Total  N (%) | Mean ± SD |
| --- | --- | --- | --- | --- | --- |
| Do you consult veterinarian or medical doctors before starting any antimicrobials for your cow and yourself respectively? | 36  48.65 | 27  36.49 | 11  14.86 | 74  100 | 1.32 ± 0.74 |
| Did you check the expiry date of any drug before using it for yourself and your animals? | 39  52.70 | 24  32.43 | 11  14.86 | 74  100 | 1.38 ± 0.73 |
| Did you complete the full course of treatment for yourself and your animals as prescribed by its professionals? | 38  51.35 | 34  45.95 | 2  2.70 | 74  100 | 1.49 ± 0.56 |
| Did you save the remaining antimicrobials for next time you and your animals getting sick? | 11  14.86 | 61  82.43 | 2  2.70 | 74  100 | 1.14 ± 0.42 |
| Do you take your cow to veterinary clinic for diagnosis of any diseases? | 38  51.35 | 25  33.78 | 11  14.86 | 74  100 | 1.36 ± 0.73 |
| Do you purchase drugs for your animals from district veterinary clinic / Pharmacy with prescri- ption? | 32  43.24 | 39  52.70 | 3  4.05 | 74  100 | 1.38 ± 0.59 |
| Do you borrow an antimicrobial from your relatives or neighbor when your cows get sick? | 3  4.05 | 46  62.16 | 25  33.78 | 74  100 | 0.69 ± 0.55 |
| Do you treat your animals with antimicrobials prescribed for humans by your decision? | 0  0.00 | 11  14.86 | 63  85.14 | 74  100 | 0.18 ± 0.42 |

SD = Standard deviation
