# Supplementary material for: Occurrence and antimicrobial susceptibility of Staphylococcus aureus in dairy farms and personnel in selected towns of West Shewa Zone, Oromia, Ethiopia
Source: PLoS One. 2022 Nov 21;17(11):e0277805. doi: 10.1371/journal.pone.0277805 (PMC9678306; doi:10.1371/journal.pone.0277805)
Supplement: Attachment — Submitted filename: Response to the reviewers.docx [file pone.0277805.s005.docx]

# **Response to the reviewers**

# The following is a point by point response given to the queries of editor and reviewers.

First, we would like to extend our sincere thanks and appreciations for all the reviewers and editors for taking their time in suggesting important comments towards improving the quality of our manuscript. In the table below we have indicated our response to each queries and accordingly incorporated the revisions in the manuscript by highlighting the changes in red for easy tracing of the reviewers. The language aspect of the manuscript was also substantially revised.

**Editor Comments to Author:**

| SN | Queries raised and section/ line number | Response given by the authors |
| --- | --- | --- |
| 1 | Please ensure that your manuscript meets PLOS ONE's style requirements, including those for file naming | Corrected as per the journals’ requirements |
| 2 | Please provide additional details regarding participant consent. In the ethics statement in the Methods and online submission information, please ensure that you have specified (1) whether consent was informed and (2) what type you obtained (for instance, written or verbal, and if verbal, how it was documented and witnessed). If your study included minors, state whether you obtained consent from parents or guardians. If the need for consent was waived by the ethics committee, please include this information. | The study participants for the questionnaire survey were briefed about the study and gave informed oral consent before the collection of the data.  In the study we didn’t involve minors |
| 3 | Please include a separate caption for each figure in your manuscript. | Captions for both figures have been included in the manuscript |
| 4 | We note you have included a table to which you do not refer in the text of your manuscript. Please ensure that you refer to Table 3 in your text; if accepted, production will need this reference to link the reader to the Table. | Table 4 was cited in the text of the revised version |

**Reviewer #1**

| SN | Queries raised and section/ line number | Response given by the authors |
| --- | --- | --- |
| 1 | The experimental design and results are well presented, however, redundancies on some sections must be addressed along with including in the discussion a few points to support the reasons why were not presented other detailed data: | Redundancies were checked and addressed.  In the discussion explanations /reasoning’s were added |
| 2 | Method used for the identification. An ISO method is available for the horizontal method for the enumeration of coagulase-positive staphylococci by counting the colonies obtained on a solid medium (Baird-Parker agar medium). Blood agar medium is not the ideal choice for the detection of *S. aureus* and other coagulase positive *Staphylococci.* | We used the Baird-Parker agar medium for colony counting. But, blood agar medium was used only for the detection of presence or absence of staphylococci in the samples and not for confirmatory purpose. |
| 3 | Detection of staphylococcal enterotoxins (SEs). Even though SEs are mentioned on the main text, there is no detection either in the milk or as gene presence in the considered strains. This could be addressed through PCR tests for the detection of at least the most known SEs. | We put the consequence staphylococcal enterotoxins (SEs) for general information. But, due to limitation of resource for molecular testing, we didn’t perform the test (it was discussed as limitation of the study) |
| 4 | Material and methods section - L115-128 please reduce the wording and the information, if necessary to support discussion, could be presented in a table instead (coordinates, msl, rainfall and so on). | It is accepted and the sentence was re-written |
| 5 | Geographical and meteorological data. The information provided in the m&m section is interesting, however, there is no contextually consideration in the discussion/ conclusion. If not relevant these information can be removed. | It is accepted and re-written |

**Reviewer #2**

| SN | Queries raised and section/ line number | Response given by the authors |
| --- | --- | --- |
| 1 | I am quite concern with the antibiotic susceptibility testing in this manuscript, I did not see the author performed any testing that can indicate whether the S. aureus strains isolated are MRSA or MSSA ? when we work with S. aureus strain, this testing should be performed because its really related with pathogenesis of this bacteria and off course with its antimicrobial susceptibility profile. Author(s) can easily identify this by testing cefoxitin or oxacillin disc. I do not think ampicillin is necessary to test in the case of S. aureus. | The comment is valid and well accepted. We did MRSA or MSSA for 21 samples by using oxacillin disc due to the inadequate number of discs we had. This result was presented in the result section as well discussed in the revised version.  In this research, we tested for ampicillin because this antibiotic is commonly used in the community of our study area. |
| 2 | Please clarify which standard reference the author(s) used on their antibiotic susceptibility testing? is it CLSI or EUCAST? | The standard we used for the antibiotic testing was CLSI, 2020 |
| 3 | The number of table in the main manuscript a bit too much. I suggest the author put some of these table in the supplementary data so the manuscript will not be too crowded | Accepted and corrected as suggested (Table 9, 10 and 11 were changed into supplementary file 1, 2 and 3 respectively). |

Reviewer #3

| SN | Queries raised and section/ line number | Response given by the authors |
| --- | --- | --- |
| 1 | Inclusion/exclusion criteria clearly explained as a separate subtopic. However, I am not sure if the authors explained why the study excluded the local lactating cows (Zebu) and opted for healthy cross breed (Zebu with Jersey and Holstein-Friesians) lactating cows. In my view, this could constitute a source of bias for the study. | In the three purposively selected towns the dairy farms are mainly managed under intensive or semi-intensive management system and the breeds farmers keep for milk purpose are exotic and their crossbred (keeping zebu cows for milk purpose in the towns was not common since they are poor in milk production). However, the issue of local lactating cows (zebu) was raised in the discussion as future areas of research |
| 2 | As a way forward, the authors can consider conducting similar study with the local lactating cows and compare the outcomes with this one. In this way the sample size will increase, and generalizability of the results can be enhanced. | Addressed in the previous response (line 522-524). |

Reviewer #4

| SN | Queries raised and section/ line number | Response given by the authors |
| --- | --- | --- |
| 1 | Abstract: Line 1: Staphylococcus aureus is one of the foodborne causing bacterial…..? Please complete the sentence with either bacterial pathogen or bacteria. There are some incomplete sentences throughout the manuscript, please make the necessary changes (e.g., lines 59-61; 65; etc.) | It is accepted and corrected |
| 2 | Introduction: Line 59-61: Staphylococcus aureus is an opportunistic bacterial pathogen with the capability to persist and multiply in a variety of environments; predominantly incriminated as one of the major causes of foodborne diseases  Line 65: and public health what? Please complete the sentence. | It is accepted and corrected |
| 3 | Methods: | |
|  | What is the rationale for choosing the towns used in the study? Why? Are those the highest milk-producing/marketing towns? Why three towns? | The three towns were purposively selected by considering the potential for dairy production, logistics and the absence of previous studies on the topic (lines 117-118). |
|  | Sample collection from milkers’ hands: What period? Before or immediately/several hours after milking? Before or after washing hands? Why? Was the method consistent throughout all sampling? Would that add variability to the results? | The milker’s hand swabs were collected consistently throughout the study immediately before milking because normally human being are the carriers of this bacteria and cross checked with cow’s milk and add variability on the results. Not only the cow but also the milkers could also be the source of contamination of raw milk, thus it might potentially add variability to the result. |
|  | Sample collection from milking buckets: When? How? Same procedure/spot for all samples? | The sample collection from milking buckets (inner wall) was done consistently before milking for all swabs. The procedures were explained under section 2.6 (Sample collection and transportations) |
|  | Cows: samples were consistently collected at the same time/period and from similar lactation stages in all cows in all the sample towns? | The samples were collected from cows of different lactation stages with the same procedure, not at the same time /period. |
| 4 | Discussion: Majority of the discussion centered around comparison of numbers/statistics between regions in and out of Ethiopia…it’s pretty difficult to keep up with all these numbers. All these may be part of the discussion, but the discussion section should mainly discuss the significance of the findings and what the results mean for the regions where the study was conducted…not just observational and reporting statistics but discussion of the relevance and significance of the findings and the study as a whole. | Accepted and addressed in detail in revised version |
|  | Line 499-506: There’s too many speculations as to why S. aureus is more prevalent in older cows than others…speculations should be limited unless there’s a direct association or probable explanation from the findings of the current study. | In the revised version the speculations were minimized and few speculation along explanations were included |
|  | Also, comparison of findings from this study with data from regions with entirely socioeconomic, cultural, and political differences seems far off, and makes the discussion bounce back and forth as to why these comparisons were made. Why? What’s the rationale of comparing data between regions? | Although the intention of comparing data between the regions of this study were to imply different risk factors, method, laboratory tests, and variability in prevalence and farmers KAPs on factors of AMR in different geographical areas, we agreed to minimize the text of discussion by eliminating comparisons. Therefore, the discussion was modified as be the suggestions given. |
|  |  |  |
|  | While revising your submission, please upload your figure files to the Preflight Analysis and Conversion Engine (PACE) digital diagnostic | Done as per the requirement |
