# Supplementary material for: Occurrence and antimicrobial susceptibility of Staphylococcus aureus in dairy farms and personnel in selected towns of West Shewa Zone, Oromia, Ethiopia
Source: PLoS One. 2022 Nov 21;17(11):e0277805. doi: 10.1371/journal.pone.0277805 (PMC9678306; doi:10.1371/journal.pone.0277805)
Supplement: Attachment — Submitted filename: Response to the reviewers 1.11.2022.docx [file pone.0277805.s006.docx]

# **Response to the reviewers**

# The following is a point by point responses given to the queries raised during the second round review process.

First, we would like to extend our sincere thanks and appreciations once again to all the reviewers and editors for taking their time in suggesting important comments towards improving the quality of our manuscript. In the table below we have indicated our response to each queries and accordingly incorporated the revisions in the manuscript by highlighting the changes in red for easy tracing. The language aspect of the manuscript was also substantially revised.

**Response to editors’ comments:**

| SN | Queries raised and section/ line number | Response given by the authors |
| --- | --- | --- |
| 1 | Journal Requirements: |  |
|  | Please review your reference list to ensure that it is complete and correct. If you have cited papers that have been retracted, please include the rationale for doing so in the manuscript text, or remove these references and replace them with relevant current references. Any changes to the reference list should be mentioned in the rebuttal letter that accompanies your revised manuscript. If you need to cite a retracted article, indicate the article’s retracted status in the References list and also include a citation and full reference for the retraction notice. | To the best of our knowledge we have revised the reference section to fit to the journal style by correcting all errors. No retracted paper was cited. |
| 2 | Additional Editor Comments: |  |
|  | Though all comments have been addressed, the manuscript needs a thorough proofreading (punctuations and spacing between words, etc.) prior to acceptance for publication. | Thank you once again for the comments. In this revised version we tried to correct such errors of spacing, punctuations, typographical, grammar, and the like. |
